# Recycling and Self-Healing of Polybenzoxazines with Dynamic Sulfide Linkages

**DOI:** 10.1038/s41598-017-05608-2

**Published:** 2017-07-12

**Authors:** Mustafa Arslan, Baris Kiskan, Yusuf Yagci

**Affiliations:** Istanbul Technical University, Department of Chemistry, 34460 Maslak Istanbul, Turkey

## Abstract

In this work, a recycling and self-healing strategy for polybenzoxazines through both S–S bond cleavage-reformation reaction and supramolecular attractions is described. Both recyclable and self-healable polybenzoxazines can be prepared from low cost chemicals with a simple procedure in only 30 minutes. For this purpose, inverse vulcanization of poly(propylene oxide)benzoxazine (PPOB) and diallybenzoxazine (B-al) with elemental sulfur was performed at 185 °C. The obtained cross-linked polymer films exhibited thermally driven recycling ability up to 5 cycles. Moreover, the self-healing ability of a test specimen was shown. Spectral characterizations, thermal stability and fracture toughness of the films were investigated after each recycling.

## Introduction

In the past few decades, polybenzoxazines (PBZs), also known as benzoxazine resins, emerged as a superior alternative to classical resol and novolac type phenolic resins. The main structural difference of PBZs from classical phenolics is the tertiary amine groups in each repeating unit, which generates immense effect especially on hydrogen bonding types and strength. Due to these unique structural characteristics, PBZs exhibit high tensile strength and modulus (100–125 MPa, and 3.8–4.5 GPa, respectively), and high glass transition temperatures (*T*_g_) (170–340 °C). Accordingly, these materials, particularly rigid ones or those admixed with transition metals, have high service temperatures, char yields, and are stable under acidic/alkali conditions. Unlike the general nature of phenolics, PBZs have low water adsorption stemming from strong intra-molecular hydrogen bonding. Another important aspect is related to their dimensional stability during synthesis. These resins are obtained by ring-opening polymerization of benzoxazine monomers at temperatures between 160–250 °C, sometimes higher, without any additive (Scheme 1)^1–6^. It should be emphasized that 1,3-isomers are the only active benzoxazines to undergo such polymerization. According to mechanistic studies, polymerization proceeds via the cationic pathway as oxazine rings have N and O atoms, which are capable of stabilizing cations during polymerization^7–9^.

**Figure 1:**
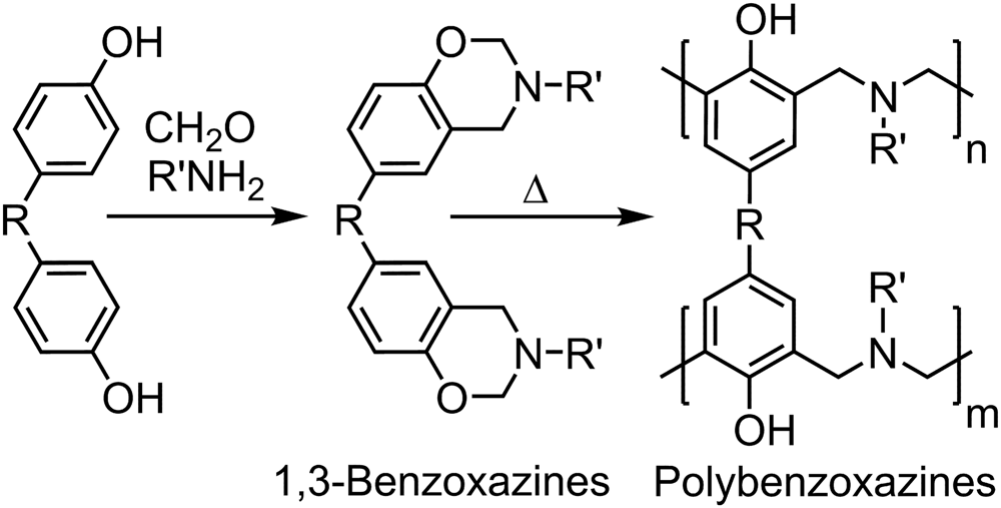
Synthesis of 1,3-benzoxazine monomer and corresponding PBZs.

The other appealing side of PBZs is the synthesis of corresponding benzoxazine monomers, which can be accomplished using any suitable phenol, a primary amine, and formaldehyde; therefore, the number of possible benzoxazines is large (Fig. 1)^10–16^. Apparently, this design flexibility brings about a huge molecular diversity and control of structure and properties of PBZs. For example, crosslinking density of the resins can be increased by introducing dimerizable or polymerizable functionalities on benzoxazines^17, 18^. The toughness of PBZs can be manipulated by using long chain amines and other soft or conversely rigid groups^19–22^. Gas forming moieties can be attached to benzoxazines causing macroporosity to occur during curing; thus, sponge-like materials can be obtained, *etc* ^23, 24^. Such suppleness in the properties of benzoxazine-based materials has led to many patents and patent applications.

Apart from monomer synthesis, combining benzoxazines with polymers as main-, side- or end-chains were extensively used as an alternative approach to modifying the properties of PBZs. Many different reactions such as hydrosilylation^25^, coupling^26–28^, Diels-Alder^29, 30^, Huisgen^31, 32^, Mannich^33–39^, esterification^40^, and addition^41–44^ *etc*. were successfully applied to obtain curable polymers bearing benzoxazines with designed properties. In recent years, research interest has been directed towards benzoxazine-based smart and advanced materials rather than alternative materials to conventional phenolics, epoxies and their composites. Although, the use of benzoxazines to produce smart materials is relatively premature, several examples such as smart coatings^45, 46^, shape memory PBZs^47^, nanofoams^48^, superhydrophobic surfaces^49–51^, self-cleaning surfaces^52^, and electrochromic devices^53, 54^ were disclosed. Furthermore, self-healing PBZ materials were produced successfully^55–57^. Adaptability of benzoxazine chemistry was also demonstrated for Li–S battery manufacturing by the reaction of benzoxazines with elemental sulfur (S_8_)^58^. Actually, the applied strategy, related to thiol-benzoxazine chemistry, was first reported by Gorodisher *et al*. and triggered synthesis of several thio-polybenzoxazines^59^. As known, sulfur is melt processable and can generate radicals on the S atom at temperatures above 160 °C. The industrially employed vulcanization of polybutadienes is based on the ability of thiyl radicals thus formed to react with double bonds. Similarly, inverse vulcanization is the treatment of molten sulfur at temperatures above 160 °C with vinylic monomers affording stable polymeric materials that could be scaled up to kilograms^60–62^. Several different materials have already been synthesized using this method ranging from IR lenses to mercury sorbents^63–67^. The process is relatively simple and sulfur can be used as much as 90%-wt. in these materials. Thus, cost effective advanced systems can easily be obtained by a simple melt process. As part of our continuous interest in developing new benzoxazine based materials to expand their applications, we now report the preparation of high sulfur containing tri-component recyclable and self-healable PBZs using inverse vulcanization chemistry. Diallyl functional benzoxazine (B-al) and main-chain PBZs were synthesized and reacted with sulfur at 185 °C. Inverse vulcanization and ring-opening polymerization took place concomitantly to produce poly(benzoxazine-*co*-sulfide) materials.

## Results and Discussions

**Figure 2:**
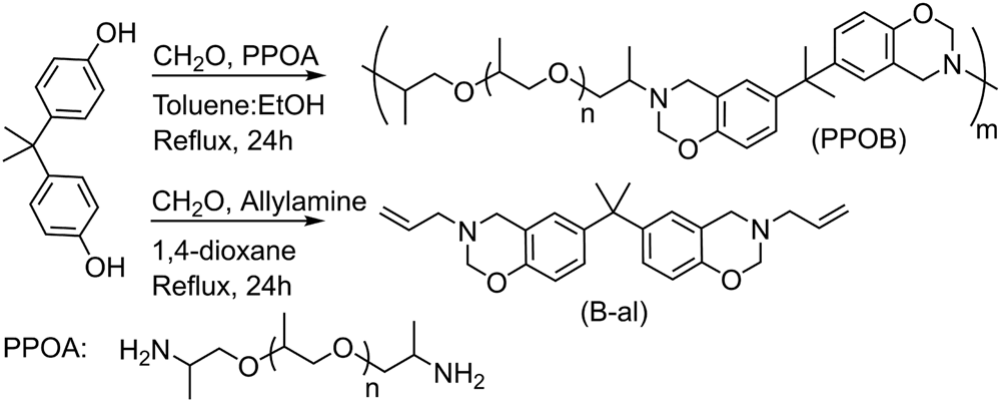
Synthesis of PPOB and B-al starting from bisphenol A.

Designing polymers that respond to temperature, light, ultrasound, pH *etc*. has been a central endeavor in polymer chemistry. Because, these polymers can significantly alter their properties in a controlled fashion by a stimulus or self-intervention, this is an important aspect for many applications. Self-healable polymers are a branch of the above-mentioned smart materials and have gained interest due to the possibility of prolonging their service life. In general, the damaged zone must have some sort of chain dynamics in a network structure to reform bonds, ensuring the healing. Consequently, a dynamic network equipped with suitable functional groups capable of performing healing reactions are required in a self-mendable material^68, 69^. Self-healable materials can also be considered as a potential recyclable polymer^70, 71^. In this study, according to the stated background, polyether amine based PBZ precursor was initially synthesized to provide a dynamic network for PBZs. Thus, poly(propylene oxide) bisamine (Jeffamine, PPOA, *M*_n_:2000), bisphenol A (BA) and paraformaldehyde were mixed and refluxed in a toluene-ethanol mixture (2:1, v/v) resulting in PBZs precursors (PPOB, *M*_n_: 12200). The other component of the recyclable/self-healing system was independently synthesized using allylamine, BA and paraformaldehyde yielding diallybisbenzoxazine (B-al) (Fig. 2).

**Figure 3:**
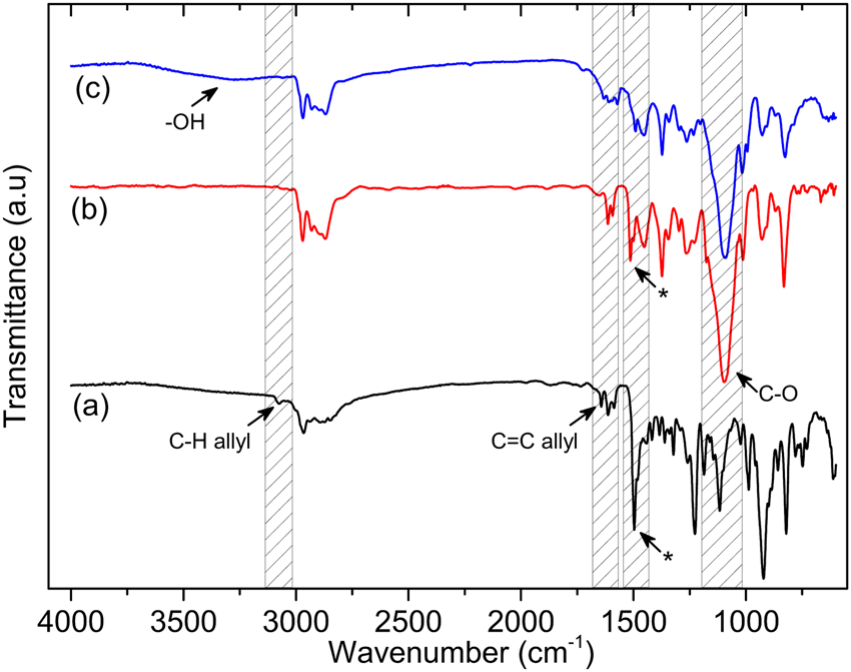
FTIR spectra of B-al (**a**), PPOB (**b**), PPOB_40_-B-al_40_-S_20_ (**c**). (*tri-substituted benzene ring of benzoxazine).

The chemical structure of the PPOB and B-al was confirmed by ^1^H NMR and FTIR spectral analysis^1^.H NMR spectra of PPOB and B-al are presented in Supplementary Figures S1 and S2, respectively. The appearance of protons resonating at 4.91, 4.84 ppm (O–CH_2_–N), and 4.02, 3.65 ppm (Ar–CH_2_–N) for PPOB and B-al is clear evidence of the benzoxazine ring formations. Moreover, the peak at 1.60 ppm (–CH_3_) and aromatic protons between 7.05–6.62 ppm disclose the bisphenol A moiety in both structures. Apart from PPOB, allyl protons of B-al are also visible at 5.92 and 5.23 ppm. Moreover, the FTIR spectra of PPOB and B-al are additional evidences for the formation of oxazine rings in both structures (Fig. 3). Allyl C–H and C=C stretching vibrations at 3074 and 1642 cm^−1^ are clearly visible. The vibration band of tri-substituted benzene ring of benzoxazine for both B-al and PPOB also appears at 1498 and 1510 cm^−1^, respectively. Besides, the C–O vibration band of PPOB verify the preservation of polyether structure after synthesis. Moreover, the stretching vibrations of aromatic C–H (3004–3062 cm^−1^) and aromatic C=C (1490–1650 cm^−1^), the out of plane bending vibrations of aromatic C–H at 930 cm^−1^ are detected for both structures.

**Figure 4:**
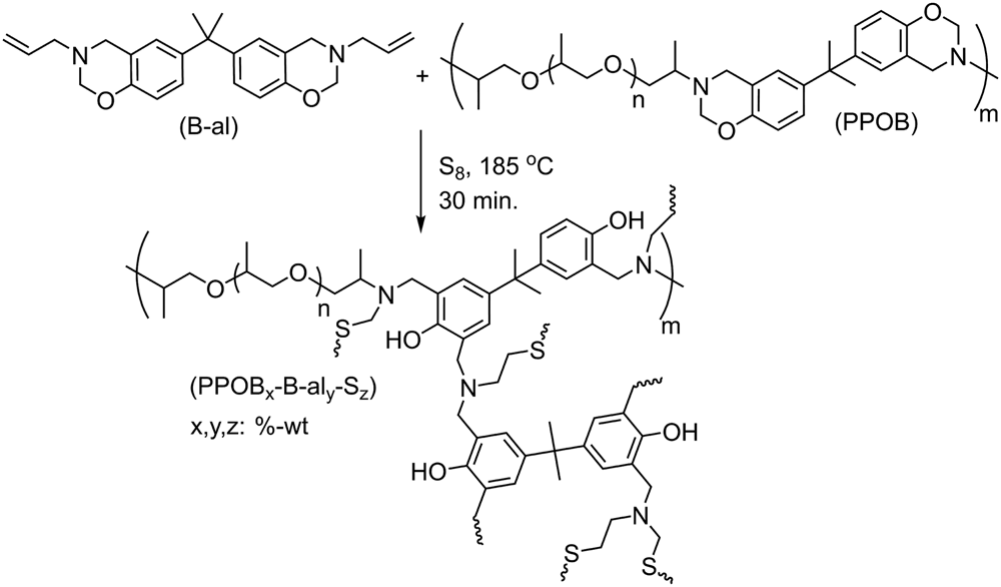
Synthesis of PPOB_x_-B-al_y_-S_z_ by using PPOB, B-al and S_8_ with various mixing ratios.

It is well-known that polysulfides are unstable at ambient conditions and degrade to form cyclooctasulfur (S_8_). However, with the inverse vulcanization approach, reacting S_8_ with vinylic monomers above 160 °C, yields stable polysulfide copolymers. The stability of such polymers was found to be great even for high sulfur contents such as 90%-wt^62^. The method is flexible as there are several different vinylic monomers affording advanced materials valuable for many applications. Previously, B-al was used as a replacement for classical vinylic monomers to obtain high sulfur containing benzoxazine resins^72^. The allyl groups readily react with sulfur radicals forming C-S covalent bonds between the benzoxazine unit and polysulfide moiety. In the meantime, ring-opening polymerization of oxazine takes place producing PBZ. This way, polysulfide is stabilized with PBZ bridges to give a copolymer. As known, the S–S bonds in polysulfide chains are dynamic and upon heating these bonds can be broken and regenerated at a certain temperature. Thus, S–S linkages are useful in designing a dynamic system such as self-healable materials. However, poly(benzoxazine-*co*-sulfide)s from B-al and S_8_ are rigid and self-healing was not observed for these samples. Therefore, in order to increase the chain mobility and improve the softness of the material, the above obtained PPOB was mixed with the B-al/S_8_ system in varying amounts. The mixture was then heated up to 185 °C to realize both curing and inverse vulcanization processes (Fig. 4) yielding copolymers, abbreviated as PPOB_x_-B-al_y_-S_z_ (x, y, z are %-wt).

The copolymers are soluble at certain mixing ratios especially at high sulfur feeds. As known, even mono functional benzoxazines form insoluble networks upon heating; the observed solubility may be due to the limited collusions of benzoxazine monomers arising from the high content of polysulfide and elemental sulfur. However, copolymers with low amounts of sulfur are insoluble.

**Figure 5:**
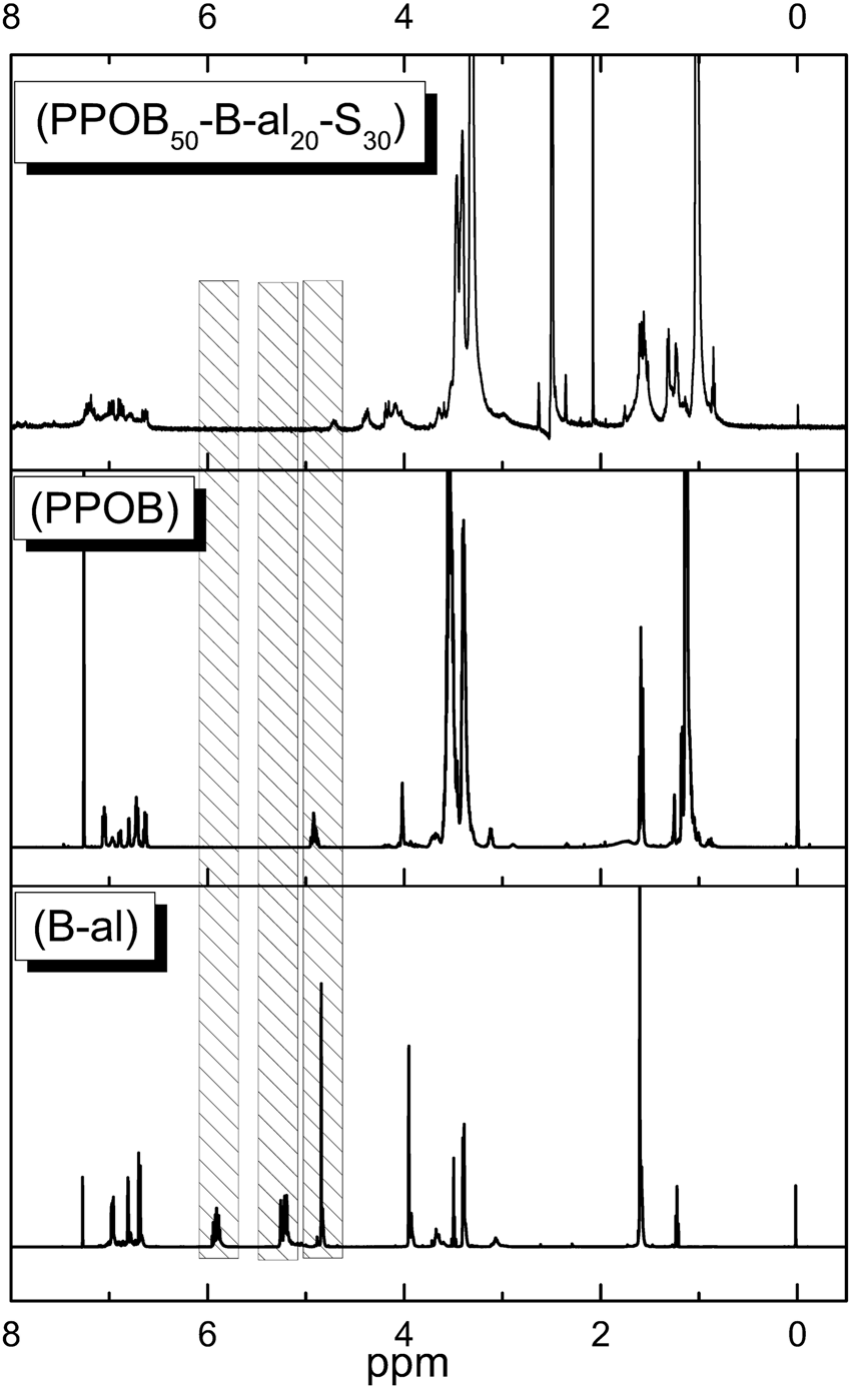
Overlaid ^1^H NMR spectra of B-al, PPOB and PPOB_50_-B-al_20_-S_30_.

The chemical structure of PPOB_x_-B-al_y_-S_z_ copolymers were confirmed using ^1^H NMR and FTIR analyses. It should be noted that for NMR analysis, soluble copolymers were prepared by increasing sulfur and PPOB content. The molecular weight of this polymer was found to be *M*_n_ = 32260 as determined by GPC. As seen in Fig. 5, when ^1^H NMR spectra of B-al, PPOB and PPOB_50_-B-al_20_-S_30_ are overlaid, the allyl protons at 5.9 (–CH=CH) and 5.2 ppm (–CH=CH) disappear after inverse vulcanization evidencing consumption of ally groups by sulfurs. Besides, the peaks that emerge at 2.5 ppm (–S–CH_2_–) after copolymerization also support the sulfur addition. Moreover, the aromatic and methyl protons of bisphenol A moiety confirm the presence of PBZ in PPOB_50_-B-al_20_-S_30_. Disappearance of N–CH_2_–O protons also shows the complete ring-opening of oxazines.

The overlaid FTIR spectra of B-al, PPOB and PPOB_40_-B-al_40_-S_20_ also reveal the consumption of both allyl groups and oxazine rings (Fig. 3). The disappearance of the allylic = C–H, C=C stretching vibrations at 3078 and 1643 cm^−1^, respectively, is clearly noted. Formation of s phenolic hydroxyl peak at 3308 cm^−1^ supports the ring-opening polymerization during the inverse vulcanization process. Moreover, the stretching vibrations of aromatic C–H (3007–3062 cm^−1^) and aromatic C=C (1560–1631 cm^−1^) and C–O ether bands, are detected for PPOB_40_-B-al_40_-S_20_. Consequently, the spectral analysis evidently supports that B-al, PPOB, and S_8_ reacted to form a network containing PBZ, polyether and polysulfide components.

**Figure 6:**
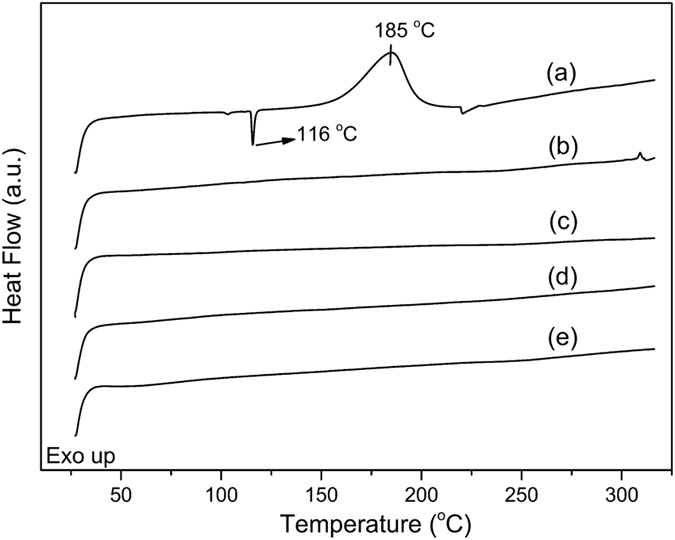
DSC thermographs of B-al, PPOB, S_8_ mixture (**a**), PPOB_40_-B-al_40_-S_20_ (**b**), 1^st^ (**c**), 3^rd^ (d), 5^th^ (**e**) healings.

In additional to spectral analysis, thermal characterization was performed to survey the benzoxazine ring structure before and after inverse vulcanization. As known, the ring-opening polymerization temperatures of benzoxazines are generally between 160–250 °C. In Supplementary Figures S3 and S4, DSC thermograms of B-al and PPOB were sketched. An onset at 203 °C, end-set at 297 °C and the curing maximum at 266 °C are detectable for B-al. Similarly, onset at 222 °C, end-set at 271 °C and a maximum at 253 °C are distinguishable for PPOB. These exotherms are drastically affected by the addition of sulfur. As seen in Fig. 6a and Table 1, the onset and the maximum curing temperatures of the B-al, PPOB, S_8_ mixture dropped down to 149 and 185 °C, respectively. The admixed S_8_ reduced the amount of exotherms compared to pristine B-al and PPOB, which can be expected as S_8_ acts as extra mass without having any exothermic process at these specific temperatures. Moreover, since the melting endotherm of S_8_ is easily detectable, it was used to track the traces of unreacted S_8_ in the final PPOB_x_-B-al_y_-S_z_ products. In Fig. 6b–e, after curing, melting endotherm of S_8_ is not noticeable for PPOB_40_-B-al_40_-S_20_ showing that sulfur is completely consumed by both allyl and oxazine groups. Similarly, curing exotherm of benzoxazines is not visible, indicating a complete curing process.

**Table 1:** DSC^a^ characteristics of B-al, PPOB and PPOB_40_-B-al_40_-S_20_.

Compound	On-set of Curing (°C)	End-set of Curing (°C)	Maximum Curing Temperature (°C)	Amount of Exotherm (J/g)
B-al^b^	203	297	266	−280
PPOB^b^	222	271	253	−20
PPOB_40_-B-al_40_-S_20_	149	206	185	−105

^a^DSC thermograms were collected under 20 mL.min^−1^ N_2_ flow and 10 °C.min^−1^ heating rate.

^b^DSC thermograms of B-al and PPOB are presented in supporting information.

In order to utilize S–S bonds incorporated into the network and provide adequate chain mobility for self-healing of PBZs a suitable admixing ratio of PPOB, B-al, and S_8_ was initially determined. The mixing ratios and copolymer properties are tabulated in Table 2. We have recognized that the S_8_ mass ratio is more critical than B-al and PPOB ratios to accomplish the ultimate aim. While the addition of excess sulfur resulted in soluble copolymers, conversely a small amount of sulfur yielded brittle, hard and insoluble materials. Moreover, B-al and S_8_ mixtures without PPOB are non-healable and depending on the S_8_ amount, these copolymers are either insoluble and rigid, or soluble. The optimum admixing weight ratios are 40% PPOB, 40% B-al and 20% S_8_ giving the PPOB_40_-B-al_40_-S_20_ copolymer. A sulfur ratio over 20% forms soluble copolymers and below this ratio, yields insoluble but highly rigid copolymers lacking required chain mobility for a self-healing system.

**Table 2:** PPOB, B-al and S_8_ feed ratios for the preparation of PPOB_x_-B-al_y_-S_z_ copolymers.

Run	PPOB (%-wt)	B-al (%-wt)	S_8_ (%-wt)	Copolymer Properties
1	80	10	10	Soft, inhomogeneous, non-healable
2	60	30	10	Rigid, inhomogeneous, non-healable
3	50	20	30	Soft, soluble, healable
4	50	30	20	Semi-soft, partially soluble
5^a^	40	40	20	Soft, insoluble, healable
6	35	50	15	Semi-soft, insoluble, partially healable
7	30	50	20	Semi-soft, partially soluble
8	30	50	0	Rigid, non-healable
9	40	40	0	Rigid, non-healable

^a^These mixing ratios yielded insoluble and self-healable material.

Typically, a self-healable film was prepared by mixing suitable amounts of PPOB, B-al and S_8_ in a Teflon mold covered with a Teflon cap tightened with clamps. The mold was placed in an open-air oven and heated up to 185 °C for 60 *min*. After cooling to room temperature, the cap was removed and the film was carefully separated from the mold. The obtained film was black in color and soft enough to cut into two pieces with scissors. After, cutting the PPOB_40_-B-al_40_-S_20_ film, the parts were kept in contact from the cut edges in a mold. The mold was then covered with a Teflon cap and the material was reheated to heal. After thermal treatment, the film reformed itself successfully (Figure S5). It should be expected that, the prepared film self-healed by S–S bond cleavage and reforming reactions after heat application. The strong hydrogen bonds of the PBZ domain might also contribute to the self-healing, as shown previously^73^. Briefly, it can be proposed that the healing mechanism is a combination of supramolecular attractions and dynamic S–S bond systems facilitated by soft segments in the structure. Moreover, another experiment was devised with a similar specimen to exhibit the recycling capacity of PPOB_40_-B-al_40_-S_20_. For this experiment, the prepared film was chopped with a knife into many parts. Then, the polymer particles were replaced in a Teflon mold and thermally treated as described for self-healing. Five cycles of chopping and healing were performed and the film successfully reformed itself after each cycle (Fig. 7 and a video as supplementary information).

**Figure 7:**
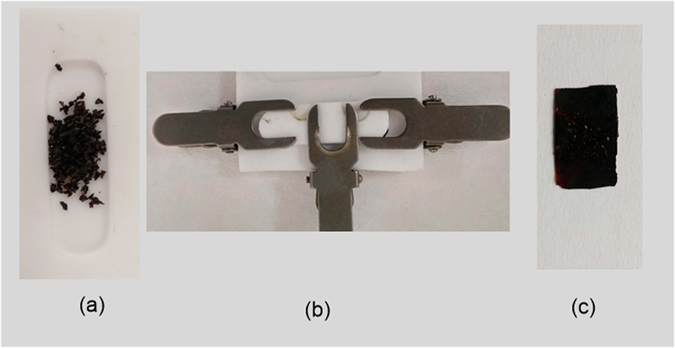
Image of chopping and 5^th^ recycling of PPOB_40_-B-al_40_-S_20_. Chopped sample (**a**), molding (**b**), after thermal treatment (**c**).

**Figure 8:**
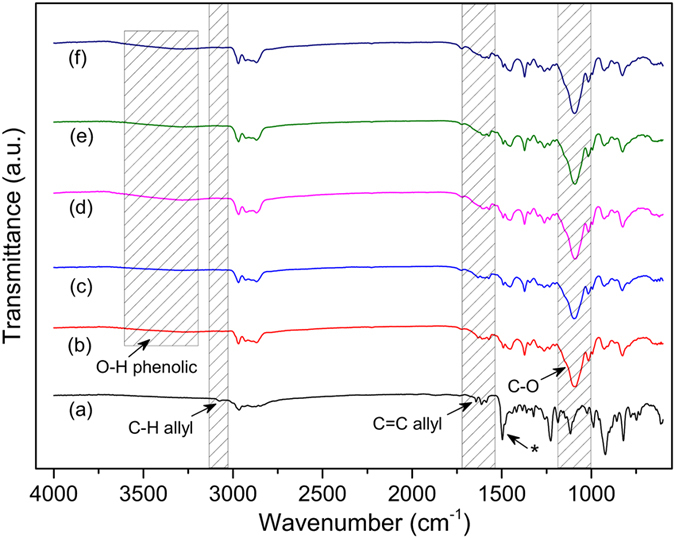
FTIR spectra of B-al (**a**), PPOB_40_-B-al_40_-S_20_ (**b**), and after 1st (**c**), 2^nd^ (**d**), 3rd (**e**), 5th (**f**) recycling processes. (*tri-substituted benzene ring of benzoxazine).

Apart from visual observations, possible changes in the structure of PPOB_40_-B-al_40_-S_20_ after cycles was monitored by thermal and spectral characterizations. DSC analysis of the film were performed after each cycle and the DSC thermograms are presented in Fig. 6. According to DSC results, polysulfide domains seem to be stable after each healing cycle and do not revert to S_8_ form which is easily detectable using the melting endotherm of sulfur. Moreover, the network preserved its main components and did not produce small fragments that can be volatile during analysis being detected as evaporation endotherms in DSC thermographs. Moreover, FTIR examinations of PPOB_40_-B-al_40_-S_20_ after each cycle did not show any distinct structural change in the material. In Fig. 8, the overlaid FTIR spectra of B-al and PPOB_40_-B-al_40_-S_20_ after healing cycles are presented. After each cycle C–O stretching vibrations of the polyether domain at 1096 cm^−1^ are visible, providing evidence that the PPOB part remained in the network after thermal treatments. Moreover, a broad phenolic O–H band at around 3308 cm^−1^ proves that hydrogen bonding was still present and the main PBZ body did not degrade even after the 5^th^ cycle. The fingerprint region comparison between FTIR spectra of B-al and PPOB_40_-B-al_40_-S_20_ indicates that the component from B-al still remained even after the final thermal treatment. Consequently, thermal and spectral inspections did not reveal any detectable structural changes for PPOB_40_-B-al_40_-S_20_ in the determination limits of DSC and FTIR.

**Figure 9:**
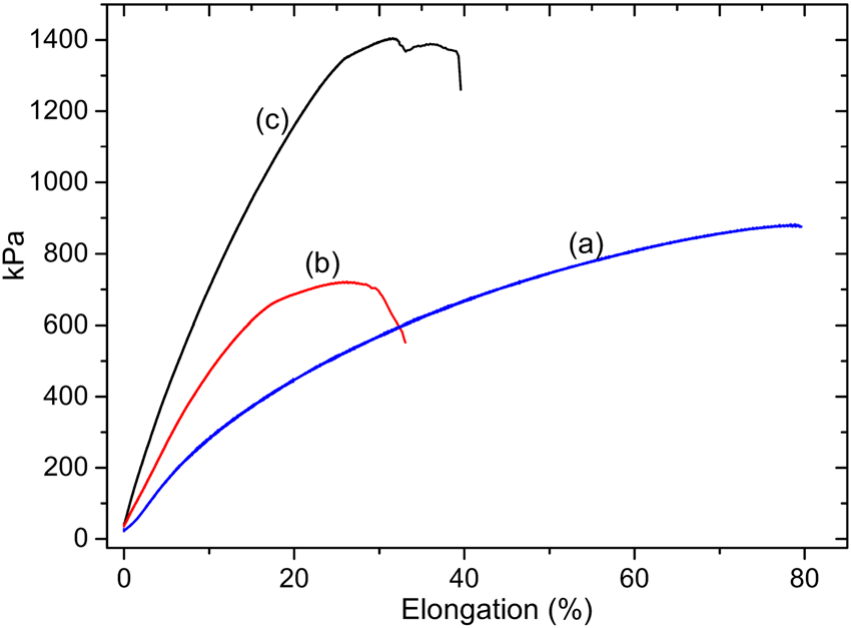
Stress-Strain (%) behavior of PPOB_40_-B-al_40_-S_20_ (**a**), cut-healed specimen (**b**), only heat treated specimen (**c**).

**Figure 10:**
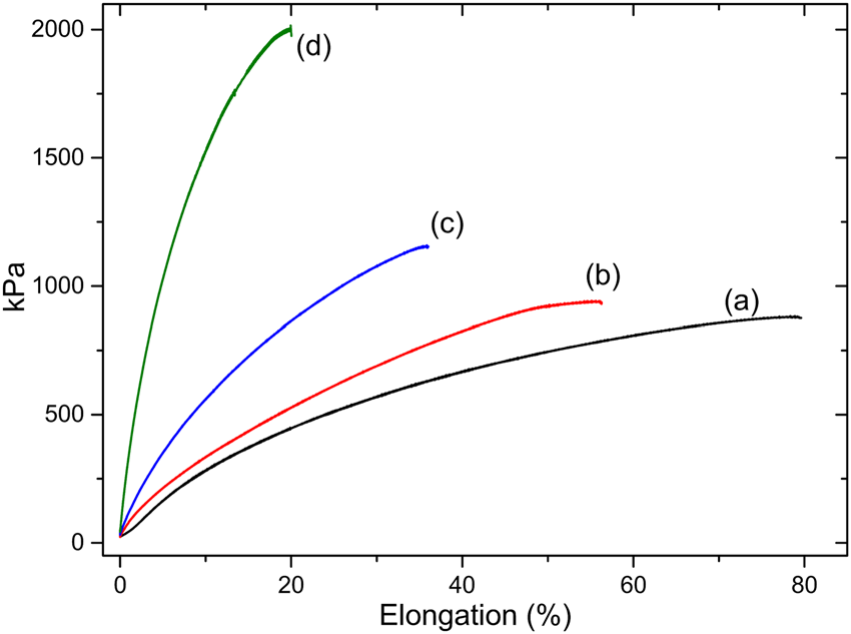
Stress-Strain (%) behavior of PPOB_40_-B-al_40_-S_20_ (**a**), 1^st^ (**b**), 3^rd^ (**c**), 5^th^ (**d**) healings.

Even though visual, thermal and FTIR analyses provide information about the recovery potential of PPOB_40_-B-al_40_-S_20_, a numeric quantification for recycling was carried out by measuring the recovery of the mechanical properties of the film (Figs 9 and 10). Although, various methods are available to quantify the extent of healing, recovery of properties such as tensile strength, fracture toughness *etc*. are generally measured. In these measurements, the “healing efficiency” of a self-healing system can be expressed as η (Eq. 1); where *K*_healed_ and *K*_virgin_ are the areas of stress-strain curve of the healed and virgin specimens^74^.

1 $${\rm{\eta }}=\frac{{K}_{healed}}{{K}_{virgin}}\times 100$$

For PPOB_40_-B-al_40_-S_20_ films, fracture toughness of the samples was measured and η was found to be ≈30 for the cut-healed sample. However, η values were found to be better for recycled samples at ≈88% for the one, ≈49% for the three, and ≈34% for the five times chopped-healed specimen. The recovery ratio of the cut-healed film is drastically smaller than the recovery of the 1^st^ recycling. This phenomenon can be explained by the contact surface area of the particles. In the recycling process, the polymer chopped into many pieces and molded. Thus, collusion of S atoms was more likely to form S–S bonds due to the increased surface area. However, for the cut-healed sample only cut edges were kept in contact and the Teflon cap of the mold applied vertical pressure on the mold, which may play a negative effect on the contact strength.

Although DSC and FTIR analysis did not reveal any structural change in PPOB_40_-B-al_40_-S_20_, the stress-strain test unveils that mechanical properties of the virgin specimen become different after each healing cycle. The toughness of the film gradually decreases and rigidity increases after heat exposure. Accordingly, the stress value for the sample changes from 877 to 2007 kPa and elongation at break reduces from 79% to 20% after the 5^th^ healing cycle. The reduction in toughness is also in accordance with healing efficiencies, since the mobility of the chains is reduced after each cycle. This phenomenon can be explained by the shortening of polysulfide chains by S–S bond cleavage and reformation reactions. Shorter chains increase the crosslinking density and at the same time rigidity which would obviously extinguish self-healing ability at a certain cycle number. On the other hand, thermal treatment of PPOB_40_-B-al_40_-S_20_ reinforces the material that is durable under ≈2000 kPa force. Thermally induced reduction of the toughness and incremental stress on the films was further investigated. In Fig. 9, the difference between PPOB_40_-B-al_40_-S_20_ and its thermal treated state is obvious. As seen, the stress value increased from 877 to 1405 kPa after 1 h thermal treatment. Also, the toughness of the film reduced ≈40% after heating, which is in accordance with the toughness reduction seen in recycled samples. In addition to mechanical analysis, we measured the weight changes of PPOB_40_-B-al_40_-S_20_ for every cycle. A weight change was observed (−1.4%) after the first healing which can be explained as the loss of sulfur presumably in the form of H_2_S gas. The formation of H_2_S during inverse vulcanization of benzoxazines was previously reported^72^. Acidic phenolic –OH groups can still lead to H_2_S formation in cured samples that are competing with the S–S reforming reaction. This possibility might be an additional explanation for chain shortening of polysulfide domains. The reduction in the amount of sulfur was also measured by elemental analysis of the samples taken from each heal cycle (Table S1). The sulfur content reduced by half at the 5^th^ cycle, compared to the initial sample. These results clearly point out that self-healable materials obtained by inverse vulcanization of acidic compounds might have a number of cycle limitations due to evaporation of sulfur, possibly in the form of H_2_S.

**Figure 11:**
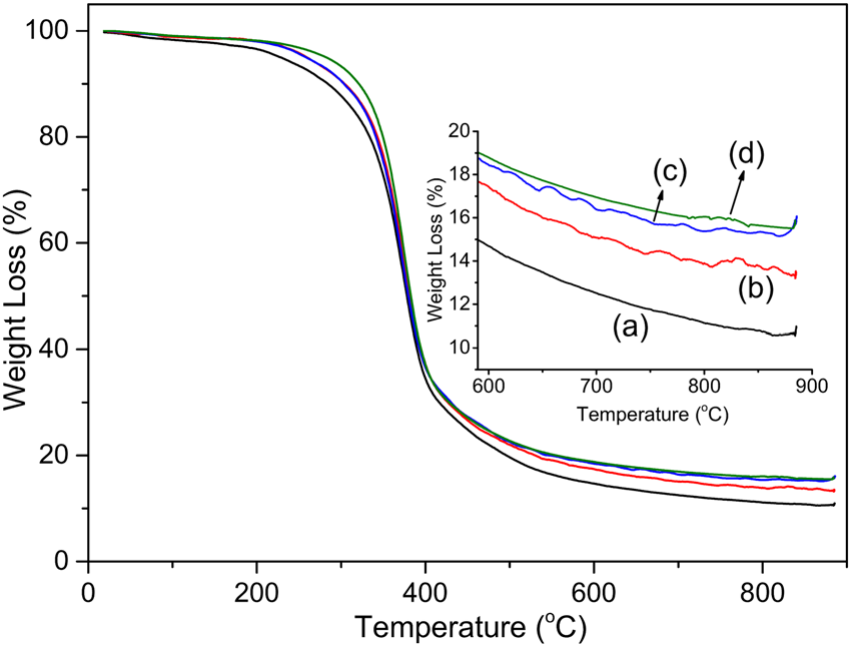
TGA traces of PPOB_40_-B-al_40_-S_20_ (**a**), 1^st^ (**b**), 3^rd^ (**c**) and 5^th^ (**d**) healing cycles.

The thermal stability of PPOB_40_-B-al_40_-S_20_ for each healing cycle was measured using thermo-gravimetric analysis (TGA). TGA traces are presented in Fig. 11 and Table 3; derivative TGA is demonstrated in Supplementary Figure S6. According to TGA data, the thermal stability of PPOB_40_-B-al_40_-S_20_ film gradually enhances towards the 5^th^ healing cycle both in terms of initial degradations and char yields. As can be seen, the PPOB_40_-B-al_40_-S_20_ sample has the lowest T_5%_, T_10%_ and T_c_ values at 230, 281 °C, 11% and after 5^th^ cycle, has the highest values at 280, 321 °C, 16%, respectively. There is a 50 °C difference for T_5%_ and 40 °C for T_10%_ showing sulfur evaporation mainly occurs in the range of 200–300 °C. Accordingly, thermal results support the chain shortening of polysulfide domains and sulfur loss in the structure. As the chains shorten and the sulfur amount is reduced, the copolymer becomes more stiff and resistant to heat. However, T_max_ is not affected by the length of polysulfide chains and the amount of sulfur in the structures. This value is the same for each cycle at ≈373 °C, indicating that the maximum weight loss is mainly dependent on degradation of PBZ domains. Generally, main weight-loss events between 300–400 °C, are assigned to amine evaporation and is the consequence of the Mannich base cleavage of PBZs. Latter weight loss can be ascribed to phenol decomposition thermal aromatization and crosslinking during degradation will finally lead to char formation.

**Table 3:** Thermal properties of the PPOB_40_-B-al_40_-S_20_ films after each healing cycle.

Film	T_5%_ (°C)	T_10%_ (°C)	T_max_ (°C)^a^	T_c_ (%)
PPOB_40_-B-al_40_-S_20_	230	281	374	11
1^st^ healing	257	306	373	14
3^rd^ healing	257	306	373	15
5^th^ healing	280	321	374	16

^a^These values extracted from derivative of TGA (Fig. S6) presented in supporting information.

T_5%_: The temperature for which the weight loss is 5%.

T_10%_: The temperature for which the weight loss is 10%.

T_max_: The temperature for maximum weight loss.

T_c_: The char yield at 800 °C.

## Conclusion

The results presented in this work demonstrate that it is possible to use inverse vulcanization of carefully designed benzoxazines to produce poly(benzoxazines-*co*-sulfide)s with self-healing and recycling property. In the approach, benzoxazine bearing PPOB were prepared through conventional main chain PBZ precursor synthesis methodology using poly(propylene oxide) amines, bisphenol A and formaldehyde to incorporate flexibility to the network. Diallybenzoxazine monomer, PPOB and elemental sulfur were mixed in suitable weight ratios and the films were prepared by thermal treatment of these components at 185 °C. Inverse vulcanization took place between sulfur atoms and allyl groups; ring-opening polymerization of the benzoxazines occurred concomitantly. The synthetic methodology described here, which can be completed within 30 *min*. by a simple melt process, is relatively fast and easy. The self-healing property of the film was demonstrated and it was shown that cross-linked films can be recyclable and heal themselves successfully up to 5 times. Evidently, the most appealing part of this strategy relies on its simplicity and the low cost of the chemicals that can be converted into advanced materials in one-pot, using the conditions outlined here.

## Methods

### Materials

4,4′-Isopropylidenediphenol (bisphenol A) (Aldrich, 97%), paraformaldehyde (Acros, 96%), poly(propylene glycol) bis(2-aminopropyl ether) (Mn ~ 2000 Da, Aldrich), allylamine (Aldrich, 98%), sulfur (S_8_, colloidal powder, reagent grade, Aldrich), sodium hydroxide (Acros, >97%), sodium sulfate (Acros, 99%), 1,4-dioxane (Aldrich, 99%), diethylether (Aldrich, ≥98%,), methanol (MeOH, Aldrich, 99%), ethanol ( ≥99.5%, Aldrich), toluene (Carlo Erba, 99.5%), chloroform (Acros, 99+%), hexane (Aldrich, 95%) were used as received.

### Measurements

All ^1^H NMR spectra were recorded on an Agilent NMR System VNMRS 500 spectrometer at room temperature in CDCl_3_ or DMSO-*d*_6_ with Si(CH_3_)_4_ as an internal standard. FTIR spectra were recorded on a Perkin-Elmer FTIR Spectrum One spectrometer. Differential Scanning Calorimetry (DSC) was performed on Perkin-Elmer Diamond DSC from 30 °C to 320 °C with a heating rate of 10 °C min^−1^. under nitrogen flow. A typical DSC sample was 2–5 mg in a 30 μL aluminum pan. Thermal gravimetric analysis (TGA) was performed on Perkin-Elmer Diamond TA/TGA with a heating rate of 10 °C min under nitrogen flow. Uniaxial elongation measurements were performed on polymeric film samples (approx. 10 mm length and 2.5 mm^2^ cross-section area). Measurements were carried out using a Perkin Elmer Pyris Diamond DMA (SII Nanotechnology Inc.) at 25 °C under 50 mN/min. load speed. The tensile strength and percentage elongation at break were recorded.

### Synthesis of poly(propylene oxide)benzoxazine (PPOB)

Bisphenol A (5.2 mmol, 1.20 g), paraformaldehyde (20.0 mmol, 0.60 g) and poly(propylene oxide) bisamine (PPO) (5 mmol, 10 g) were dissolved with 100 mL of toluene and 50 mL of ethanol and refluxed for 24 h in a 500 mL round bottomed flask equipped with a condenser. The solvent was evaporated under vacuum and a blondish oily product was precipitated in cold n-hexane. The precipitation process was done two times. The polymer was dried under vacuum for 1 day.

### Synthesis of 6,6′-(propane-2,2-diyl)bis(3-allyl-3,4-dihydro-2H-benzo[e][1,3]oxazine) (B-al)

In a 500 mL round bottomed flask, allyamine (30.8 g, 0.54 mol) in 1,4-dioxane (200 mL) was cooled in an ice bath. Paraformaldehyde (32.4 g, 1.08 mol) was added portion-wise to this solution over 10 min. Thereafter, bisphenol A (61.6 g, 0.27 mol) was added to the solution and the mixture was refluxed for 24 h. After this reaction time, the solvent was evaporated using a rotary evaporator. The resulting oily product was dissolved in diethyl ether (250 mL) and extracted with 0.1 N sodium hydroxide various times to remove unreacted bisphenol A. Then, to neutralize the solution, diethyl ether solution washed with distilled water (80 mL) for two times. Afterwards, the diethyl ether layer was dried with anhydrous Na_2_SO_4_ and filtered. Solvent was evaporated under vacuum and the remaining oily product was dissolved in MeOH (30 mL). Water was added drop by drop into the MeOH solution until it became turbid, and then refrigerated (4 °C). The precipitated sticky mass was obtained by decantation of MeOH and washing with excess water. The product was dried under vacuum at 60 °C for 24 h. (Yield, 63%)

### General procedure for the preparation of poly(benzoxazine-*co*-sulfide) (PPOB_x_-B-al_y_-S_z_)

To a 20 mL test tube, equipped with a magnetic stir bar, PBOB, B-al and sulfur were added. The tube was heated up to 185 °C with vigorous stirring in an oil bath. In the first 5 minutes a clear orange solution formed. Then, the color of the solution changed to brown-black. The overall reaction time was 30 *min*. in total. After cooling the tube to r.t., it was broken and the product was collected. In these reactions, sulfur feed ratios were 10, 15, 20 and 30-wt% while the other components’ feed ratios were 30, 35, 40, 50, 60, 80-wt% (see Table 2). A similar procedure was applied for film preparations; instead of glass tubes, Teflon molds with Teflon covers were used. The mixture dispersed in mold uniformly and the tightly covered mold was placed in an open-air oven and heated up to 185 °C for 60 *min*. After cooling, the cap was removed and the film was separated from the Teflon mold.

## Electronic supplementary material

Supplementary Video

Supplementary Information

## References

[1] Ghosh NN, Kiskan B, Yagci Y (2007). Polybenzoxazines - New high performance thermosetting resins: Synthesis and properties. Prog. Polym. Sci..

[2] Takeichi T, Kawauchi T, Agag T (2008). High Performance Polybenzoxazines as a Novel Type of Phenolic Resin. Polym. J..

[3] Nair CPR (2004). Advances in addition-cure phenolic resins. Prog. Polym. Sci..

[4] Ishida, H. in *Handbook of Benzoxazine Resins* 3-81 (Elsevier, 2011).

[5] Arslan, M., Kiskan, B. & Yagci, Y. In *Encycl. Polym. Sci. Technol*. (John Wiley & Sons, Inc., 2015).

[6] Wang J, Xu YZ, Fu YF, Liu XD (2016). Latent curing systems stabilized by reaction equilibrium in homogeneous mixtures of benzoxazine and amine. Sci. Rep..

[7] Wang YX, Ishida H (1999). Cationic ring-opening polymerization of benzoxazines. Polymer.

[8] Kasapoglu F, Cianga I, Yagci Y, Takeichi T (2003). Photoinitiated cationic polymerization of monofunctional benzoxazine. J. Polym. Sci. Part A: Polym. Chem..

[9] Hamerton I (2013). Examining the Initiation of the Polymerization Mechanism and Network Development in Aromatic Polybenzoxazines. Macromolecules.

[10] Oie H, Sudo A, Endo T (2010). Acceleration effect of N-allyl group on thermally induced ring-opening polymerization of 1,3-benzoxazine. J. Polym. Sci. Part A: Polym. Chem..

[11] Wang C, Sun J, Liu X, Sudo A, Endo T (2012). Synthesis and copolymerization of fully bio-based benzoxazines from guaiacol, furfurylamine and stearylamine. Green Chem..

[12] Wang C (2013). Synthesis and thermal properties of a bio-based polybenzoxazine with curing promoter. J. Polym. Sci. Part A: Polym. Chem..

[13] Kawaguchi AW, Sudo A, Endo T (2014). Thiol-functionalized 1,3-benzoxazine: Preparation and its use as a precursor for highly polymerizable benzoxazine monomers bearing sulfide moiety. J. Polym. Sci. Part A: Polym. Chem..

[14] Andreu R, Reina JA, Ronda JC (2008). Studies on the thermal polymerization of substituted benzoxazine monomers: Electronic effects. J. Polym. Sci. Part A: Polym. Chem..

[15] Imran M, Kiskan B, Yagci Y (2013). Concise synthesis and characterization of unsymmetric 1,3-benzoxazines by tandem reactions. Tetrahedron Lett..

[16] Sini NK, Bijwe J, Varma IK (2014). Renewable benzoxazine monomer from Vanillin: Synthesis, characterization, and studies on curing behavior. J. Polym. Sci. Part A: Polym. Chem..

[17] Agag T, Takeichi T (2003). Synthesis and characterization of novel benzoxazine monomers containing allyl groups and their high performance thermosets. Macromolecules.

[18] Takeichi T, Nakamura K, Agag T, Muto H (2004). Synthesis of cresol-based benzoxazine monomers containing allyl groups and the properties of the polymers therefrom. Des. Monomers Polym..

[19] Kiskan B, Koz B, Yagci Y (2009). Synthesis and Characterization of Fluid 1,3-Benzoxazine Monomers and Their Thermally Activated Curing. J. Polym. Sci. Part A: Polym. Chem..

[20] Baranek AD (2012). Flexible aliphatic-bridged bisphenol-based polybenzoxazines. Polym. Chem..

[21] Allen DJ, Ishida H (2006). Physical and mechanical properties of flexible polybenzoxazine resins: Effect of aliphatic diamine chain length. J. Appl. Polym. Sci..

[22] Kudoh R, Sudo A, Endo T (2010). A Highly Reactive Benzoxazine Monomer, 1-(2-hydroxyethyl)-1,3-Benzoxazine: Activation of Benzoxazine by Neighboring Group Participation of Hydroxyl Group. Macromolecules.

[23] Zuniga C (2011). Polybenzoxazines from Renewable Diphenolic Acid. J. Polym. Sci. Part A: Polym. Chem..

[24] Zúñiga C, Lligadas G, Ronda JC, Galià M, Cádiz V (2012). Self-foaming diphenolic acid benzoxazine. Polymer (United Kingdom).

[25] Aydogan B, Sureka D, Kiskan B, Yagci Y (2010). Polysiloxane-Containing Benzoxazine Moieties in the Main Chain. J. Polym. Sci. Part A: Polym. Chem..

[26] Demir KD, Kiskan B, Yagci Y (2011). Thermally Curable Acetylene-Containing Main-Chain Benzoxazine Polymers via Sonogashira Coupling Reaction. Macromolecules.

[27] Demir KD, Kiskan B, Latthe SS, Demirel AL, Yagci Y (2013). Thermally curable fluorinated main chain benzoxazine polyethers via Ullmann coupling. Polym. Chem..

[28] Chernykh A, Agag T, Ishida H (2009). Effect of Polymerizing Diacetylene Groups on the Lowering of Polymerization Temperature of Benzoxazine Groups in the Highly Thermally Stable, Main-Chain-Type Polybenzoxazines. Macromolecules.

[29] Chou C-I, Liu Y-L (2008). High performance thermosets from a curable Diels–Alder polymer possessing benzoxazine groups in the main chain. J. Polym. Sci. Part A: Polym. Chem..

[30] Wang Y-H, Chang C-M, Liu Y-L (2012). Benzoxazine-functionalized multi-walled carbon nanotubes for preparation of electrically-conductive polybenzoxazines. Polymer.

[31] Nagai A (2008). Synthesis and crosslinking behavior of a novel linear polymer bearing 1,2,3-triazol the main chain by and benzoxazine groups in a step-growth click-coupling reaction. J. Polym. Sci. Part A: Polym. Chem..

[32] Ye YS, Huang YJ, Chang FC, Xue ZG, Xie XL (2014). Synthesis and characterization of thermally cured polytriazole polymers incorporating main or side chain benzoxazine crosslinking moieties. Polym. Chem..

[33] Demir KD, Kiskan B, Aydogan B, Yagci Y (2013). Thermally curable main-chain benzoxazine prepolymers via polycondensation route. React. Funct. Polym..

[34] Hanbeyoglu B, Kiskan B, Yagci Y (2013). Hydroxyl Functional Polybenzoxazine Precursor as a Versatile Platform for Post-Polymer Modifications. Macromolecules.

[35] Bektas S, Kiskan B, Orakdogen N, Yagci Y (2015). Synthesis and properties of organo-gels by thiol-benzoxazine chemistry. Polymer.

[36] Chernykh A, Liu JP, Ishida H (2006). Synthesis and properties of a new crosslinkable polymer containing benzoxazine moiety in the main chain. Polymer.

[37] Takeichi T, Kano T, Agag T (2005). Synthesis and thermal cure of high molecular weight polybenzoxazine precursors and the properties of the thermosets. Polymer.

[38] Ates S (2011). Synthesis, characterization and thermally activated curing of polysulfones with benzoxazine end groups. Polymer.

[39] Lin CH (2012). Flexible polybenzoxazine thermosets with high glass transition temperatures and low surface free energies. Polym. Chem..

[40] Kiskan B, Yagci Y, Ishida H (2008). Synthesis, characterization,, and properties of new thermally curable polyetheresters containing benzoxazine moieties in the main chain. J. Polym. Sci. Part A: Polym. Chem.

[41] Semerci E, Kiskan B, Yagci Y (2015). Thiol reactive polybenzoxazine precursors: A novel route to functional polymers by thiol-oxazine chemistry. Eur. Polym. J..

[42] Kawaguchi AW, Sudo A, Endo T (2012). Polymerization–Depolymerization System Based on Reversible Addition-Dissociation Reaction of 1,3-Benzoxazine with Thiol. ACS Macro Lett..

[43] Arslan M, Kiskan B, Yagci Y (2016). Post-Modification of Polybutadienes by Photoinduced Hydrogen Abstraction from Benzoxazines and Their Thermally Activated Curing. Macromolecules.

[44] Kiskan B, Yagci Y, Sahmethogilu E, Toppare L (2007). Preparation of conductive polybenzoxazines by oxidative polymerization. J. Polym. Sci. Part A: Polym. Chem..

[45] Liao C-S, Wang C-F, Lin H-C, Chou H-Y, Chang F-C (2009). Fabrication of Patterned Superhydrophobic Polybenzoxazine Hybrid Surfaces. Langmuir.

[46] Aydogan C, Kiskan B, Hacioglu SO, Toppare L, Yagci Y (2014). Electrochemical manipulation of adhesion strength of polybenzoxazines on metal surfaces: from strong adhesion to dismantling. RSC Adv..

[47] Sarawut R, Montha L, Kasinee H, Pornnapa K, Isala D (2013). Shape memory polymers from benzoxazine-modified epoxy. Smart Mater. Struct..

[48] Cheng H (2016). Super flame-retardant lightweight rime-like carbon-phenolic nanofoam. Sci. Rep.

[49] Wang C-F (2006). Low-Surface-Free-Energy Materials Based on Polybenzoxazines. Angew. Chem. Int. Edit..

[50] Liu J, Lu X, Xin Z, Zhou C (2013). Synthesis and Surface Properties of Low Surface Free Energy Silane-Functional Polybenzoxazine Films. Langmuir.

[51] Kuo S-W, Wu Y-C, Wang C-F, Jeong K-U (2009). Preparing Low-Surface-Energy Polymer Materials by Minimizing Intermolecular Hydrogen-Bonding Interactions. J. Phys. Chem. C.

[52] Zhang W, Lu X, Xin Z, Zhou C (2015). A self-cleaning polybenzoxazine/TiO2 surface with superhydrophobicity and superoleophilicity for oil/water separation. Nanoscale.

[53] Lin L-C (2014). Novel near-infrared and multi-colored electrochromic polybenzoxazines with electroactive triarylamine moieties. J. Mater. Chem. C.

[54] Shih H-K, Chu Y-L, Chang F-C, Zhu C-Y, Kuo S-W (2015). A cross-linkable triphenylamine derivative as a hole injection/transporting material in organic light-emitting diodes. Polym. Chem..

[55] Kiskan B, Yagci Y (2014). Self‐healing of poly (propylene oxide)‐polybenzoxazine thermosets by photoinduced coumarine dimerization. J. Polym. Sci. Part A: Polym. Chem..

[56] Taskin OS, Kiskan B, Yagci Y (2013). Polybenzoxazine Precursors As Self-Healing Agents for Polysulfones. Macromolecules.

[57] Arslan M, Kiskan B, Yagci Y (2015). Benzoxazine-Based Thermosets with Autonomous Self-Healing Ability. Macromolecules.

[58] Ghosh A, Shukla S, Khosla GS, Lochab B, Mitra S (2016). Sustainable Sulfur-rich Copolymer/Graphene Composite as Lithium-Sulfur Battery Cathode with Excellent Electrochemical Performance. Sci. Rep..

[59] Gorodisher, I., DeVoe, R. J. & Webb, R. J. in Handbook of Benzoxazine Resins (ed Hatsuo IshidaTarek Agag) 211-234 (Elsevier, 2011).

[60] Lian Q, Li Y, Li K, Cheng J, Zhang J (2017). Insights into the Vulcanization Mechanism through a Simple and Facile Approach to the Sulfur Cleavage Behavior. Macromolecules.

[61] Griebel JJ, Li G, Glass RS, Char K, Pyun J (2015). Kilogram scale inverse vulcanization of elemental sulfur to prepare high capacity polymer electrodes for Li-S batteries. J. Polym. Sci. Part A: Polym. Chem..

[62] Chung WJ (2013). The use of elemental sulfur as an alternative feedstock for polymeric materials. Nat. Chem..

[63] Griebel JJ (2015). Dynamic Covalent Polymers via Inverse Vulcanization of Elemental Sulfur for Healable Infrared Optical Materials. ACS Macro Lett..

[64] Griebel JJ (2014). New Infrared Transmitting Material via Inverse Vulcanization of Elemental Sulfur to Prepare High Refractive Index Polymers. Adv. Mater..

[65] Simmonds AG (2014). Inverse Vulcanization of Elemental Sulfur to Prepare Polymeric Electrode Materials for Li–S Batteries. ACS Macro Lett..

[66] Arslan M, Kiskan B, Cengiz EC, Demir-Cakan R, Yagci Y (2016). Inverse vulcanization of bismaleimide and divinylbenzene by elemental sulfur for lithium sulfur batteries. Eur. Polym. J..

[67] Crockett MP (2016). Sulfur-Limonene Polysulfide: A Material Synthesized Entirely from Industrial By-Products and Its Use in Removing Toxic Metals from Water and Soil. Angew. Chem. Int. Edit..

[68] Wool RP (2008). Self-healing materials: a review. Soft Matter.

[69] Wu DY, Meure S, Solomon D (2008). Self-healing polymeric materials: A review of recent developments. Prog. Polym. Sci..

[70] Xu WM, Rong MZ, Zhang MQ (2016). Sunlight driven self-healing, reshaping and recycling of a robust, transparent and yellowing-resistant polymer. J. Mater. Chem. A.

[71] Xiang HP, Rong MZ, Zhang MQ (2016). Self-healing, Reshaping, and Recycling of Vulcanized Chloroprene Rubber: A Case Study of Multitask Cyclic Utilization of Cross-linked Polymer. ACS Sustainable Chemistry & Engineering.

[72] Arslan M, Kiskan B, Yagci Y (2016). Combining Elemental Sulfur with Polybenzoxazines via Inverse Vulcanization. Macromolecules.

[73] Kim HD, Ishida H (2002). A study on hydrogen-bonded network structure of polybenzoxazines. J. Phys. Chem. A.

[74] Brown EN, Sottos NR, White SR (2002). Fracture testing of a self-healing polymer composite. Exp. Mech..

