# Supplementary material for: Recycling and Self-Healing of Polybenzoxazines with Dynamic Sulfide Linkages
Source: Sci Rep. 2017 Jul 12;7:5207. doi: 10.1038/s41598-017-05608-2 (PMC5507923; doi:10.1038/s41598-017-05608-2)
Supplement: Supplementary file 2 — Supplementary Information [file 41598_2017_5608_MOESM2_ESM.doc]

**Supplementary Information**

RECYCLING AND SELF-HEALING OF POLYBENZOXAZINES WITH DYNAMIC SULFIDE LINKAGES

Mustafa Arslan^1^, Baris Kiskan*^,1^, Yusuf Yagci*,^1^

*^1^Istanbul Technical University, Department of Chemistry, 34460, Maslak, Istanbul, Turkey*

|  |
| --- |
| **Figure S1:** ^1^H NMR spectrum of PPOB precursor |

|  |
| --- |
| **Figure S2:** ^1^H NMR spectrum of B-al monomer |

|  |
| --- |
| **Figure S3:** DSC thermograph of B-al |

| 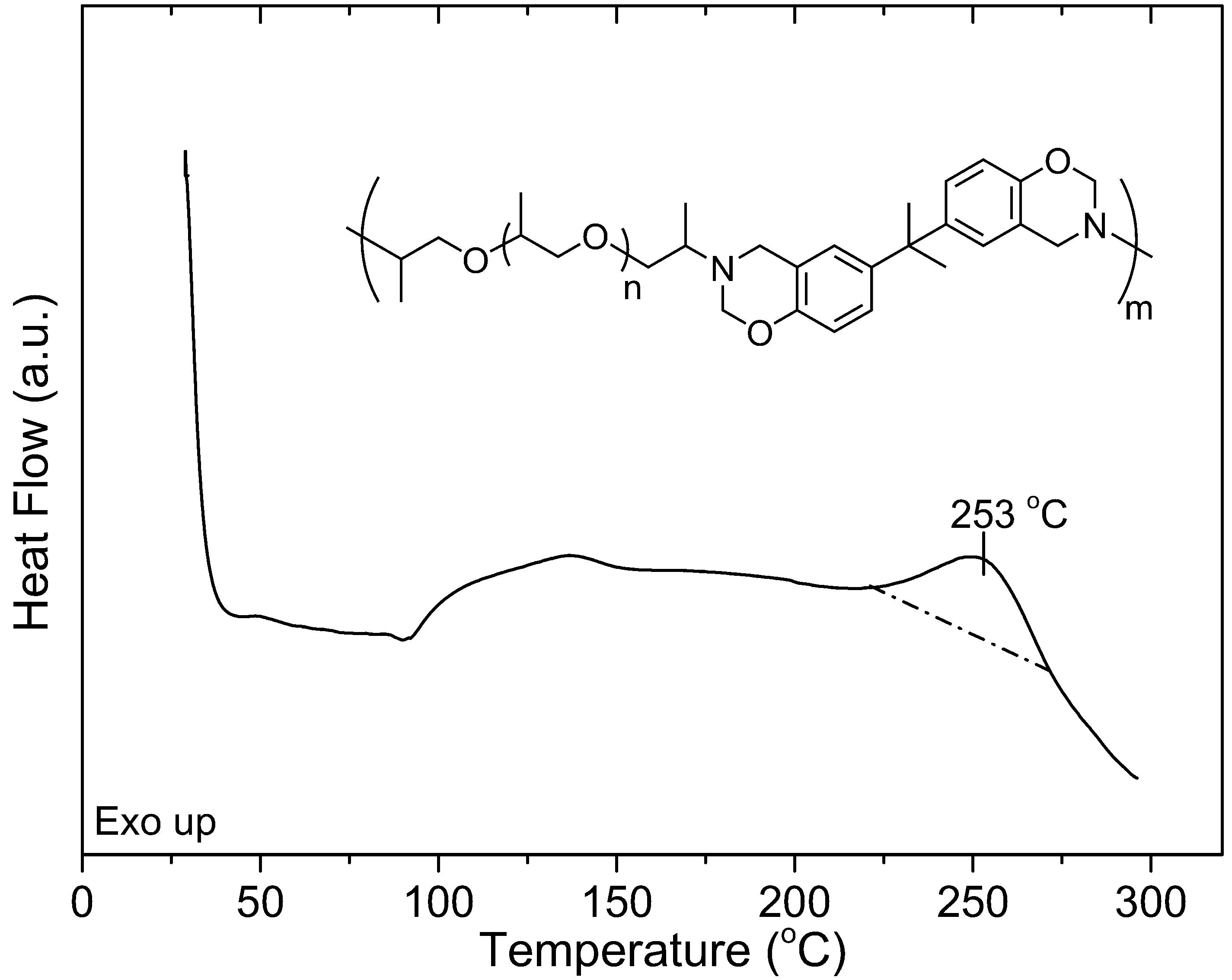 |
| --- |
| **Figure S4:** DSC thermograph of PPOB |

|  |
| --- |
| **Figure S5:** Images of cut and healed specimen. |

**Table S1:** Elemental analysis results of PPOB_40_-B-al_40_-S_20_ for each healing cycle.

| **Cycles** | **S%** | **C%** | **H%** |
| --- | --- | --- | --- |
| 1^st^ | 4.55 | 59.61 | 5.08 |
| 3^rd^ | 3.82 | 59.15 | 4.99 |
| 5^th^ | 2.22 | 59.10 | 4.95 |

|  |
| --- |
| **Figure S6:** Derivative TGA traces of PPOB_40_-B-al_40_-S_20_ (a), 1^st^ (b), 3^rd^ (c) and 5^th^ (d) healing cycles. |
